# Exploiting the DNA Damage Response for Prostate Cancer Therapy

**DOI:** 10.3390/cancers16010083

**Published:** 2023-12-23

**Authors:** Travis H. Stracker, Oloruntoba I. Osagie, Freddy E. Escorcia, Deborah E. Citrin

**Affiliations:** 1 Radiation Oncology Branch, Center for Cancer Research, National Cancer Institute, National Institutes of Health, Bethesda, MD 20892, USA; toba.osagie@nih.gov (O.I.O.); freddy.escorcia@nih.gov (F.E.E.); citrind@mail.nih.gov (D.E.C.); 2 Molecular Imaging Branch, Center for Cancer Research, National Cancer Institute, National Institutes of Health, Bethesda, MD 20892, USA

**Keywords:** prostate cancer, androgen, DNA damage response, radiotherapy, radiopharmaceutical therapy, PARP, ATR, ATM, DNA-PK, immunotherapy, hypoxia

## Abstract

**Simple Summary:**

Localized prostate cancer has a favorable prognosis and can be effectively treated with radiotherapy, as well as therapies that target hormone production and signaling. However, if the disease progresses past these treatments, treatment options are limited, and the disease often becomes fatal. In this review, we discuss the changes in treatment as prostate cancer develops, as well as the genetic mutations that accompany advanced prostate cancer. We highlight how these mutations can provide clinical opportunities to manipulate the DNA damage response for therapeutic gain using cancer-specific alterations in the genome and new therapeutic agents that are under development or entering clinical trials.

**Abstract:**

Prostate cancers that progress despite androgen deprivation develop into castration-resistant prostate cancer, a fatal disease with few treatment options. In this review, we discuss the current understanding of prostate cancer subtypes and alterations in the DNA damage response (DDR) that can predispose to the development of prostate cancer and affect its progression. We identify barriers to conventional treatments, such as radiotherapy, and discuss the development of new therapies, many of which target the DDR or take advantage of recurring genetic alterations in the DDR. We place this in the context of advances in understanding the genetic variation and immune landscape of CRPC that could help guide their use in future treatment strategies. Finally, we discuss several new and emerging agents that may advance the treatment of lethal disease, highlighting selected clinical trials.

## 1. Introduction

Prostate cancer (PCa) is the second most common cancer in men and the second leading cause of cancer-related death amongst men worldwide [1]. The clinical diagnosis staging, and prognostic stratification of PCa relies on digital rectal examination, the histological analysis of tumor biopsies with grade grouping using the Gleason scoring system, and serum prostatic specific antigen (PSA) levels [2]. The majority of PCa patients are diagnosed with low-risk localized disease and require minimal intervention at diagnosis, with active surveillance considered a favored approach. Radical prostatectomy or radiotherapy (RT), in the form of either external beam RT (EBRT) or internal RT via brachytherapy, are the major treatment options for intermediate-risk localized PCa, with surveillance offered to patients with favorable risk factors and low-volume disease [3]. Patients diagnosed with high-risk localized disease are treated with RT with androgen deprivation therapy (ADT) or surgery to provide local control and reduce the risk of recurrence or metastasis [2]. In the setting of metastatic disease at presentation, systemic therapy with ADT and next-generation androgen receptor (AR) signaling inhibitors (ARSIs), such as enzalutamide, are a first-line therapy to prevent the reactivation of AR signaling [4].

**Figure 1:**
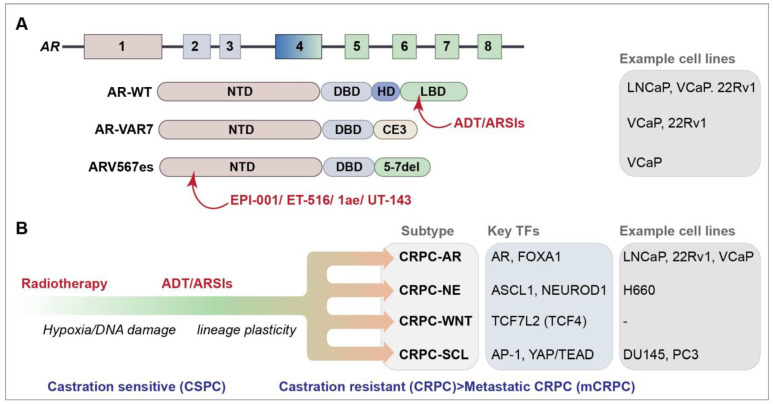
(**A**) Schematic of the *AR* gene and AR and selected AR-V protein domains. Protein domains are described in the text and color coded to the exon schematic. Selected agents that target protein domains are indicated in red (see text for further details) [18,19,20,21]. AR-VAR7 arises due to the splicing to cryptic exon 3 (CE3) and ARV567es from the deletion of exons 5–7; cell lines that express each of the selected genes endogenously are listed [6]. (**B**) Progression of PCa and identified CRPC subtypes are depicted [22]. Key transcription factors (TFs) and some commonly used cell lines representing the subtypes are shown.

The AR is a master transcription factor (TF) that facilitates the effects of androgens on target tissues and is a major target in PCa therapy [5]. The AR is comprised of four domains: an intrinsically disordered N-terminal domain (NTD) that is essential for activating transcription, a DNA-binding domain (DBD) that recognizes androgen response elements (AREs) in target genes, a hinge domain (HD) and a ligand-binding domain (LBD) that binds androgen (Figure 1A). The LBD is the target of frontline ADT and ARSI therapies that have had a strong impact on the control of castration-sensitive PCa (Figure 1A). Extensive research efforts have focused on the role of AR-dependent resistance mechanisms in PCa, which play vital roles in disease progression and treatment resistance. These mechanisms include AR amplification and overexpression, the expression of AR mutants and overexpression of AR variants (AR-Vs) that often lack the LBD (Figure 1A) [6]. Over 20 AR-Vs have been identified, but the functional relevance of many of these to disease progression remains unclear (reviewed in references [7,8]). Some AR-Vs, including AR-V7 and AR-V567es, are produced at the protein level and have constitutive transcriptional activity that can drive proliferation in the absence of androgens. The presence of AR-V7 is a clinical biomarker of resistance to AR-targeted therapies; however, its direct clinical targeting is hampered by the lack of the LBD targeted by ARSIs (Figure 1A) [9,10,11,12,13,14,15].

While many tumors are initially responsive to RT, ADT, and ARSIs, around 20–30% of patients will progress within 2–3 years and eventually transition to a more aggressive, AR-independent form of PCa known as castration-resistant prostate cancer (CRPC) or metastatic CRPC (mCRPC), both of which are incurable and often lethal within 2 years [16,17].

The genomic landscape of CRPC, as well as the full extent of its heterogeneity remains unclear. Lineage plasticity, facilitated in part by defects in chromatin regulation, plays a significant role in treatment resistance in CRPC [23,24]. Recent work has revealed four CRPC subtypes based on the chromatin accessibility and gene expression profiles of patient-derived cell lines and organoids (Figure 1B) [22,24]. Two known subtypes were identified: the AR-dependent subtype (CRPC-AR), characterized by AR amplifications or mutations and the AR gene signature, and the neuroendocrine (NE) subtype (CRPC-NE), characterized by the expression of NE markers, such as *SYP* and *CHGA* [22]. Two new subtypes were defined: the WNT-dependent subtype (CRPC-WNT), which featured alterations in the WNT pathway genes, such as *CTNNB1* (also referred to as beta-catenin), and the stem cell-like subtype (CRPC-SCL), defined by the enrichment of mammary stem cell signature genes and the high expression of the cancer stem cell markers *CD44* and *TACSTD2*.

Hypoxia is a prominent feature of the tumor microenvironment (TME) proposed to promote lineage plasticity, increase DNA damage, and drive treatment resistance in cancers, including PCa [24,25,26]. Hypoxia is a major form of cellular stress, driving cells to adapt through transcriptional and molecular changes, including a decrease in proliferation, heightened glycolysis, the modulation of DNA repair factors, and angiogenesis [27]. Tumor hypoxia is characterized by low tissue oxygenation, ranging from 0.3% to 4.2% oxygen (2–32 mmHg) and PCa has been determined to have low oxygenation levels compared to tumors from other tissues [28,29,30,31]. In response to hypoxia, tumor cells regulate distinct physiological responses through hypoxia-inducible factors (HIFs) [27]. Tumor hypoxia can have several biological and clinical consequences in PCa, including elevated genomic instability, increased mutational burden, castration resistance, an increased likelihood of metastasis, chemoresistance, and radioresistance [31,32,33,34].

RT is a central and effective treatment modality for localized PCa. Hypoxic conditions reduce the efficacy of RT by preventing the oxygen-dependent “fixation” of damage caused by free radicals [35]. Hypoxic signaling can also contribute to the abnormal vascularization of tumors, limiting drug delivery and contributing to the reduced radiosensitivity and chemosensitivity of tumor cells [35]. Moreover, hypoxia contributes to an immunosuppressive TME and represents a challenge to immunotherapy [36]. Substantial efforts have been made to identify strategies to overcome hypoxia-induced therapeutic resistance (reviewed in reference [36]). These include agents that enhance oxygen delivery to radiosensitize hypoxic cells, electron-affinic radiosensitizers and hypoxia-activated prodrugs (HAPs). However, these approaches have yet to make a major clinical impact in PCa treatment.

**Figure 2:**
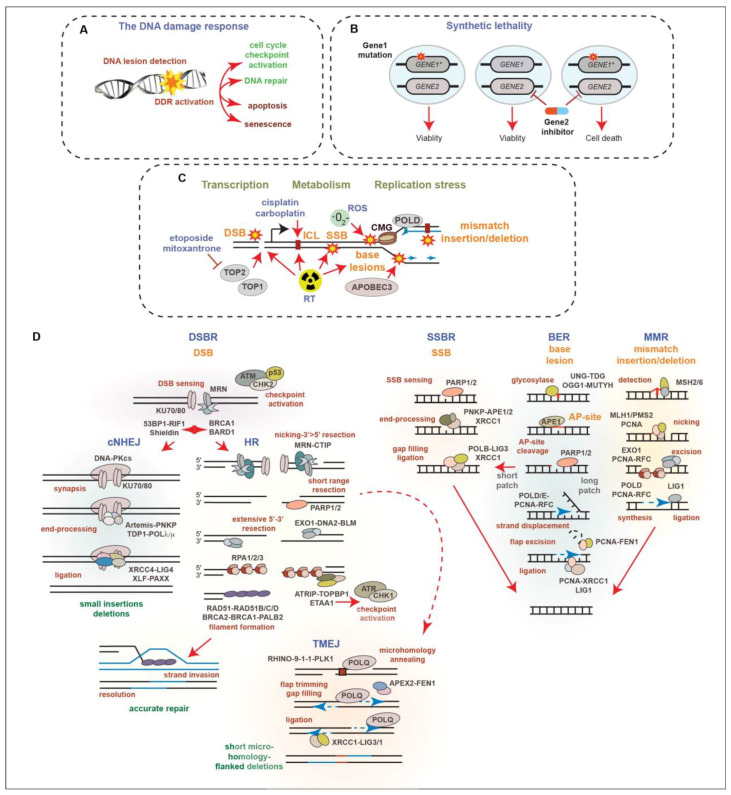
(**A**) Schematic of the DNA damage response. DNA lesions are sensed, activating signal transduction networks that trigger cell cycle checkpoints and DNA repair, as well as cell fate decisions like apoptosis and senescence. (**B**) Example of synthetic lethality. Targeting *GENE2* is tolerated by normal cells but is toxic to cancer cells due to a dependency on the function of *GENE1* that is mutated (indicated by star) in the cancer cell. (**C**) Schematic of the major sources of DNA damage (indicated by star) in PCa. Endogenous DSBs arise due to topoisomerase activity during transcription and through replication stress: the slowing or stalling of replication forks and accumulation of ssDNA. Genotoxic treatments used in therapy, including RT, topoisomerase 2 inhibitors (etoposide, mitoxantrone) and platinum agents, such as cisplatin and carboplatin, cause base lesions, DNA interstrand strand crosslinks (ICL), SSBs, and DSBs through distinct mechanisms. (**D**) Summaries of selected DNA lesions (orange) and repair pathways (blue). Major factors (black) and steps (red) of double-strand break repair (DSBR), single-strand break repair (SSBR), base excision repair (BER) and mismatch repair (MMR) are depicted. Connections between DSBR pathways, classical non-homologous end joining (cNHEJ), homologous recombination (HR), and polymerase theta mediated end joining (TMEJ) are shown. Nucleotide excision repair, ICL repair, and DNA–protein crosslink repair details are omitted for brevity. See text and references for additional information and perspectives [41,42,43,44,45,46,47,48,49,50,51].

Ionizing radiation induces DNA damage in the form of a variety of base lesions, single-strand breaks (SSBs), double-strand breaks (DSBs), which are considered the most toxic DNA lesion, and DNA crosslinks [35]. The success of RT and many chemotherapies is strongly influenced by the status of the DNA damage response (DDR), a signal transduction network that identifies and responds to many different types of DNA lesions (Figure 2A) [37,38]. The DDR triggers changes in cellular proliferation, through the activation of cell cycle checkpoints, and regulates the DNA repair machinery to resolve DNA lesions and protect genome integrity. Depending on the cell type or the extent of DNA damage, the DDR can also activate cell death or senescence pathways to promote the removal of cells with potentially oncogenic DNA damage. The DDR acts as an inducible barrier to oncogenesis, creating a selective pressure to lose the key components of DDR signaling that allow for tumor growth through the evasion of tumor-suppressive mechanisms, including DNA repair, cell cycle checkpoint arrest, and cell fate signaling pathways [39]. Defects in the DDR result in genomic instability, a hallmark of cancer, in the forms of DNA amplifications and deletions, translocations, and mutations [40]. As the disease advances, the progressive loss of DDR genes can accelerate tumor growth and DNA damage tolerance. However, this can also enhance the efficacy of some DNA-damaging treatments, as recurrent gene losses can provide opportunities to take advantage of synthetic lethal interactions (Figure 2B) that can be targeted using specific inhibitors or RT [41].

There is an unmet critical need for new therapeutic modalities for CRPC and mCRPC and several promising strategies taking advantage of recurring genetic alterations, particularly those affecting the DNA damage response (DDR), have entered clinical trials. This review will focus on the current barriers to the treatment of CRPC and new developments in therapeutic approaches that act by causing DNA damage, taking advantage of mutations in the DDR or modulating the DDR to improve treatment responses.

## 2. The DNA Damage Response in Prostate Cancer

Several types of endogenous DNA damage arise in PCa due to cellular processes, including metabolism, transcription, and DNA replication (Figure 2C). These include mismatched bases, base insertions or deletions due to polymerase errors, as well as base lesions caused by reactive oxygen species (ROS), spontaneous deamination, or the activities of the Apolipoprotein B mRNA editing enzyme, catalytic polypeptide (APOBEC) family of enzymes that deaminate cytosines [44,45,52]. SSBs can arise due to the processing of base lesions and DSBs can be caused by the activity of topoisomerase enzymes acting during transcription and replication, the processing of stalled replication forks during replication stress or after collisions with transcriptional machinery, and the mitotic breakage of under-replicated DNA regions [42,46,49]. RT introduces many types of base lesions, DNA crosslinks, and SSBs that can elevate replication stress levels, as well as DSBs that trigger cell death or senescence pathways. Compared to many other cancer types, few DNA-damaging agents aside from RT are used in the treatment of PCa, as localized PCa is well-controlled with RT and CRPC is generally poorly responsive to genotoxic compounds, potentially due to slow growth rates. Genotoxic agents, including topoisomerase-2 inhibitors, such as etoposide and mitoxantrone, as well as platinum DNA crosslinking agents, like cisplatin, are used in palliative care, as well as the treatment of CRPC-NE in accordance with the guidelines for small cell lung cancer [2]. The use of taxanes, which poison microtubules to prevent proliferation, has largely overtaken mitoxantrone used in selected settings in CRPC treatment, but acquired resistance remains a significant issue [53,54,55,56].

DNA double-strand breaks (DSBs) activate a number of kinases, including ATM, ATR, and DNA-PKcs, as well as additional effector kinases such as CHK1 and CHK2, which together, regulate cell cycle checkpoints, DNA repair, and cell fate decisions (Figure 2D) [37]. ATM is activated directly by the detection of DSBs by the MRE11-RAD50-NBS1 (MRN) complex, and subsequently activates CHK2 that in turn modifies many of the same targets as ATM. The accumulation of ssDNA, which can arise due to the nucleolytic resection of DSBs, replication stress (replication fork stalling and ssDNA accumulation), or by gaps that can form behind the replication fork, leads to the activation of ATR and CHK1 [57]. ATR activation is triggered by ssDNA coated with RPA and the activities of the ATRIP, TOPBP1, and ETAA1 proteins. Like ATM/CHK2, ATR/CHK1 plays a key role in regulating cell cycle checkpoints, as well as multiple DNA repair factors and cell fate pathways.

The two major pathways of DSB repair are classical nonhomologous end-joining (cNHEJ) and homologous recombination (HR) (Figure 2D) [42]. These pathways compete for DNA ends, with cNHEJ functioning throughout the cell cycle and HR operating in S and G2 due to the need for a sister chromatid template to guide accurate recombinational repair. DNA-PKcs, along with KU70 and KU80, form the DNA-dependent protein kinase that plays a central role in promoting cNHEJ of DSBs. KU70/80 recognizes DNA ends, promoting the activation of DNA-PKcs, as well as the recruitment of the Artemis nuclease, and other factors, which can clean up blocked ends, allowing for their ligation by LIG4 and additional factors, including XRCC4, XLF, and PAXX [42,43]. Short insertions and deletions at the breakpoint can occur as a result of cNHEJ repair. A protein complex that includes 53BP1, RIF1, and Shieldin (SHLD1/2/3 and REV7) is critical for preventing the access of BRCA1-BARD1 and multiple nucleases that resect DNA ends to promote HR-mediated repair. The MRN complex can introduce nicks near the break via its endonuclease activity and resect 3′-5′, generating ends that release KU70/80 and are incompatible with cNHEJ-mediated ligation. Subsequent 5′-3′ resection by multiple nucleases and helicases, including EXO1, DNA2, and BLM, generates the overhangs needed for stand invasion and the HR-mediated repair of DSBs. Overhangs are bound by the tripartite RPA (RPA1/2/3) that is exchanged for RAD51, as well as its paralogs, RAD51A/B/C/D, to facilitate HR, resulting in accurate repair. Additional alternative end-joining pathways can contribute to DSB repair and DNA damage tolerance [42,43]. These include polymerase theta-mediated end-joining (TMEJ) (also referred to as alternative end joining or microhomology-mediated end-joining). TMEJ uses microhomology exposed by limited resection to anneal and modify ends, fill gaps, and ligate ends through the activities of PARP1/2, POLQ (Polymerase theta), XRCC1, and LIG3, resulting in short microhomology-flanked deletions (Figure 2D) [37,41]. Recent work has demonstrated that TMEJ can operate during mitosis in homologous recombination-deficient (HRD) cells in conjunction with RHINO, the 9-1-1 complex, and PLK1, as well as the MDC1-CIP2A-TOPBP1 proteins that tether mitotic DNA breaks [58,59,60,61,62].

Base and nucleotide lesions can be processed by several excision mechanisms, which include single-strand base repair (SSBR) and base excision repair (BER) (Figure 2B). The Poly (ADP-ribose) polymerases, particularly PARP1 and PARP2, play important roles in regulating these pathways [48,49]. PARP1/2 activity promotes the recruitment of additional factors, including XRCC1. The processing of some base lesion types leads to SSBs and unrepaired SSBs can result in DSBs, through replication fork progression or replication–transcription collisions. In addition to SSBR and BER, MMR also processes base lesions that include base mismatches, insertions, and deletions that are generated by the replicative polymerases. MMR is critical to prevent mutagenesis and the loss of MMR is particularly relevant in many cancer types including PCa, as it increases microsatellite instability (MSI) and tumor mutational burden (TMB) [63]. The increased mutational load can lead to oncogene activation or tumor suppressor inactivation, for example through mutations in *KRAS* or *TP53*. Multiple single base pair mutational signatures for MMR deficiency (dMMR) have been identified, including SBS6 and SBS15, as well as numerous doublet-base substitution and indel signatures [64].

High TMB can also occur due to mutations in several polymerases that affect proofreading capabilities, including *POLE* and *POLD1*, as well as the APOBEC3 cytidine deaminases that can be activated by replication stress [52,65,66]. The APOBEC3-mediated deamination of cytosines to uracil causes mispairing during DNA replication or the subsequent processing of uracil by glycosylases, such as UNG, generates abasic sites (AP-sites) that can elevate TMB [52]. APOBEC3A and APOBEC3B are the prominent mutagenic drivers in several cancer types, giving rise to a distinct mutational signature (SBS2 and SBS13) that can be distinguished from dMMR and identified by cancer genome sequencing and analysis [67,68,69,70].

While only a single hypermutagenic polymerase mutation has been reported in CRPC to our knowledge, both dMMR and APOBEC signatures appear in CRPC, but occur infrequently compared to some other cancer types [71,72,73]. MSI-high/dMMR tumors were identified in some CRPC cohorts, constituting up to 5% of analyzed patients depending on the cohort analyzed [74,75]. Numerous prostate cancer samples exhibit APOBEC signature mutations that increase along the spectrum of disease progression from localized to mCRPC, although the overall frequency is low compared to several other tumor types [71,76]. Recent work has also suggested that APOBEC3B activity in CRPC may have an important role in mutagenesis that can facilitate lineage plasticity and ARSI resistance (see Section 6 for additional details) [77].

Germline mutations in DNA repair genes affecting multiple pathways have been implicated in PCa susceptibility. These are estimated to occur in ~5–15% of total PCa cases, with around 12% occurring in mCRPC, where several are associated with high-grade disease. Germline mutations in DDR genes primarily occur in genes involved in cell cycle checkpoint signaling (*ATM*~2%, *CHEK2*~2.8%, TP53~0.7%), HR (*BRCA2*~4.8%, *BRCA1*~1.3%, *PALB2*~0.6%, *NBN*~0.3%, *RAD50*~0.3%, *RAD51*~3%, *RAD51C*~0.2% and *RAD51D* < 0.2%), BER (*MUTYH*~2.4%), and MMR (*MSH2*~0.7%, *PMS2*~0.5%, *MSH6*~0.5%, *MLH1*~0.06%) [78,79]. Somatic mutations in the DDR genes occur in localized PCa (~11%) and increase in frequency as disease progresses, with DDR mutations estimated to occur in 20% or more of mCRPC cases [80,81,82,83,84]. These include *ATM* (~7%), *BRCA2* (~10%), and *BRCA1* (0.5%), accounting for around 20% of the DDR mutations, as well as *ATR* (~8%), *RAD51* (~2%), *PARP1* (~6%), *MLH1* (~1.4%), and *MSH2* (~3%). The high frequency of DDR mutations in CRPC represents a potential opportunity for exploiting synthetic lethalities or targeted therapies in patients that progress from RT and anti-androgen therapies.

## 3. New Approaches for Targeting AR-Variants

ARSIs, such as Enzalutamide, are effective at targeting AR function through the competitive binding of its LBD (Figure 1A). However, the emergence of AR-Vs that lack the LBD, through mutations or cryptic splicing, confer resistance to ADT and ARSIs in CRPC [9,85]. One prominent variant, AR-V7, enhances the expression of DNA repair genes and confers radioresistance in various PCa cell lines, indicating that targeting AR-Vs could not only address the resistance to ARSIs, but also augment RT and potentially other therapies targeting the DDR [86,87].

Screening for small molecules that inhibited the transcriptional activity of the intrinsically disordered AR NTD led to the identification of EPI-001, the first compound to directly target this domain (Figure 1A) [18]. Recent studies have described new molecules, some derived from EPI-001, which target the AR or AR-V NTD (Figure 1A) [18,20,21,88]. These new molecules disrupt the liquid–liquid phase separation (LLPS) propensity, protein–protein interactions and transcriptional activity of AR and AR-Vs and showed anti-tumor effects in animal models, including those expressing AR-Vs. While the additional development of these compounds will be required for clinical use, they further demonstrate that targeting the intrinsically disordered domain of the AR and AR-Vs is a feasible strategy for impairing its function in tumorigenesis. These agents will also allow for the testing of combination strategies with other agents being explored in CRPC, including PARP inhibitors that also impair AR and AR-V transcriptional activity, albeit indirectly [89,90]. The approach of targeting intrinsically disordered domains could be employed against other TFs in future work, potentially guided by the recent subtype classification that identified specific TFs associated with AR-negative/low CRPCs (Figure 1B) [22]. In addition, as many DNA repair proteins contain intrinsically disordered domains and undergo LLPS, these approaches could potentially be used to directly target a number of key DNA repair factors, including 53BP1 and TOPBP1, in future therapies [91].

## 4. PARP Inhibitors in CRPC

The seminal discovery that HRD was synthetically lethal with PARP inhibitors ignited interest in targeting PARP activity across many cancer types [92,93]. The low toxicity of PARP inhibitors, as well as the loss of heterozygosity in germline HRD mutations or somatic mutations in cancer cells, but not surrounding tissues, makes PARP inhibitor lethality with HRD attractive due to its tumor-selective effects. Several potent PARP inhibitors were developed and approved for clinical use and multiple mechanisms have been proposed to explain the synthetic lethal interaction between PARP inhibitors and HRD, which has been reviewed extensively elsewhere [37,94]. In brief, the generation of DSBs due to the combined impairment of BER and HR, toxic ssDNA gaps behind the replication fork, the incorporation of ribonucleotides that can trap PARP, and the degree of PARP1/2 trapping, have all been proposed to contribute to the toxicity of PARP inhibitors in different settings. Extensive CRISPR screening has identified many additional modifiers of PARP inhibitor toxicity and notable resistance mechanisms, which in some cases, appear to be specific to the particular HR mutation [94,95,96]. A prominent example is the loss of 53BP1, RIF1, or members of the Shieldin complex that rescues PARP inhibitor sensitivity resulting from the loss of *BRCA1* (Figure 2B) [95].

PARP inhibitor clinical trials for prostate cancer were recently reviewed and analyzed extensively, so here, we will summarize several major trials for four PARP inhibitors that have been tested in mCRPC: Olaparib, Rucaparib, Niraparib, and Talazaporib [97,98,99]. The important takeaways overall are that PARP inhibitors appear to be safe, although anemia and fatigue are observed in some patients, and in biomarker-selected populations, PARP inhibitors are beneficial for patients. However, there remains a need for a more detailed understanding of how specific genomic alterations influence PARP inhibitor efficacy.

The TOPARP (NCT01682772), PROfound (NCT02987543), and TRITON2 (NCT02952534) trials tested the two currently FDA-approved PARP inhibitors Olaparib (Lynparza) and Rucaparib (Rubraca) as monotherapies in mCRPC with HRD [100,101,102,103,104]. The TOPARP trial tested Olaparib monotherapy on an unselected patient population that had progressed on ARSIs or taxanes and found a higher response rate in patients with DDR mutations, including all patients with *BRCA2* mutations and the majority of those with *ATM* mutations [105]. The PROfound trial selected patients that had progressed following ARSI treatment and that had HRD, defined either by *BRCA1*, *BRCA2*, or *ATM* mutations, or a panel of another 12 DDR related genes. The trial showed that Olaparib monotherapy was more effective than ARSI treatment, measuring radiographic progression-free survival and overall survival [100,102]. The TRITON2 trial selected patients that progressed on previous ARSI or taxane treatment and had mutations in *BRCA1/2*, *ATM*, or additionally selected DDR genes [101,103,104]. Improved objective response rates (ORRs) to Rucaparib were observed, particularly for *BRCA2*-deficient patients, and differences in responses based on the specific DDR gene altered were also notable. The loss of *PALB2*, *FANCA*, *BRIP1*, and *RAD51B* were associated with improved responses, while responses were limited in *ATM-*, *CHEK2-*, and *CDK12*-deficient cancers [103].

The Phase II GALAHAD (NCT02854436) and TALAPRO-1 (NCT03148795) trials recently provided compelling evidence for the efficacy of the PARP inhibitors Niraparib and Talazaporib in mCRPC [106,107]. The GALAHAD trial assessed patients post-ARSI or -taxane treatment, using two cohorts that had either *BRCA1/2* alterations or alterations in a panel of other DDR genes including *ATM*, *BRIP1*, *CHEK2*, *FANCA*, *HDAC2*, or *PALB2*, with improved ORRs observed in both, but with the *BRCA1/2* cohort showing the most dramatic responses to Niraparib [106]. The TALAPRO-1 trial again assessed patients post-ARSI or -taxane treatment, using a panel of DDR-related genes for the selection of a single cohort [107]. The carriers of *BRCA2* alterations showed the most dramatic responses, but tumors with alterations in *BRCA1*, *PALB2*, and *ATM* also showed partial or complete responses.

Despite its identification as a PARP inhibitor sensitizer in multiple settings, *ATM* loss in PCa does not appear to consistently confer a strong benefit to PARP inhibitor-treated patients, in contrast to the loss of *BRCA2* that was consistently associated with the highest benefit to patients treated with multiple PARP inhibitors [98,108,109,110,111,112,113,114]. The extent of PARP trapping was demonstrated to correlate with PARP inhibitor toxicity, with Talazaparib being the most potent PARP inhibitor tested, followed by Niraparib and Rucaparib/ Olaparib [115,116]. The differing conclusions of the TRITON2 and TALAPRO-1 trials regarding *ATM* may suggest that the extent of PARP trapping is important for the synthetic interaction with *ATM* loss. Alternatively, it could reflect the effects of the specific ATM alleles on the overall function of ATM or rely on other, unknown co-mutations. All four of these PARP inhibitors, as well as another PARP inhibitor, Veliparib, continue to be tested in additional Phase 1–3 trials in combination therapies with ARSIs, Temozolomide, and corticosteroids in patients with HRD [99]. As additional clinical data emerges, the ability to both select responsive patient populations, as well as effective drug combinations, will no doubt improve the clinical outcomes of mCRPC.

While many genes associated with HRD are currently screened for in CRPC patients entering clinical trials, the full repertoire of genes that could benefit PARP inhibitor treatment remains to be identified. Recent work in cell lines and mouse models has demonstrated that the loss of *MMS22L* and *RNASEH2B*, which both occur frequently in CRPC (~7% and ~12% deep deletions in mCRPC, respectively), may also improve PARP inhibitor efficacy [83]. The loss of MMS22L, or its binding partner TONSL, which work together to facilitate RAD51 deposition in HR, were shown to sensitize PCa cell lines to PARP inhibitors [117,118,119]. It is notable that the deletion of *MMS22L* or *TONSL* failed to sensitize two CRPC-SCL cell lines, DU145 and PC3, to PARP inhibitors, potentially due to their lack of functional TP53 signaling [120]. The investigators further demonstrated that the deletion of *CHEK2* (encoding CHK2) suppressed PARP inhibitor sensitivity due to the loss of *BRCA2* suppression by CHK2-TP53-E2F7. Functional CHK2-TP53 is not required for the synthetic interaction between *BRCA1/2* loss and PARP inhibitors, so it remains to be seen if other genetic suppressors may be relevant in the context of *MMS22L* deficiency in CRPC tumors [92,121].

**Figure 3:**
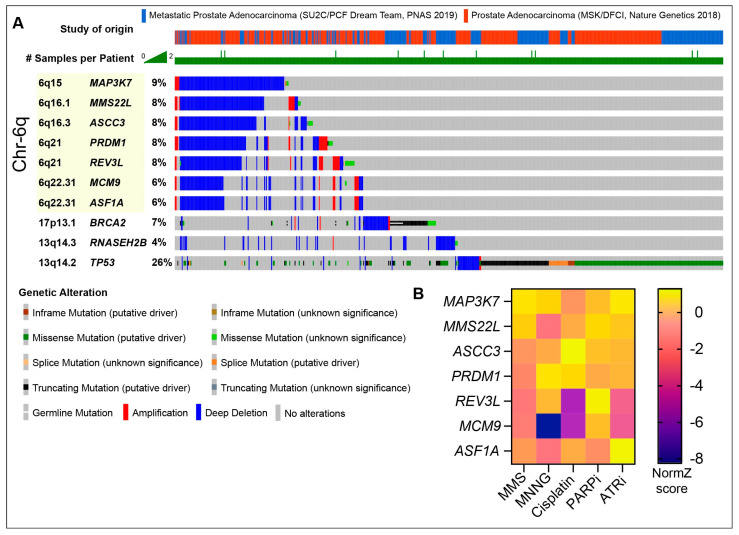
(**A**) Oncoprint of selected DDR genes in the recurrent chromosome 6q deletion in samples from two PCa cohorts; SU2C/PCF Dream Team (mCRPC) and MSK/DFCI (Prostate Adenocarcinoma) [83,123,124,125,143]. Selected DDR genes that are typically lost in the 6q deletion and their relative locations are highlighted (see text for details). PARP sensitizers *BRCA2* and *RNASEH2B* are shown for comparison, along with common driver gene *TP53*. Note that the 6q locus deletions show mutual exclusivity with *TP53* (cbioportal.org). Patient samples without alterations in the selected genes are omitted for brevity. (**B**) Sensitivity (NormZ-score) to selected agents in RPE1-hTERT-Cas9-TP53-/- cells in CRISPR screens [127]. PARPi = Olaparib and ATRi = AZD6738.

*MMS22L* deletions appear to occur in PCa tumors primarily in the context of a large recurrent deletion in chromosome 6q, potentially driven by *PRDM1* loss (Figure 3A) [122]. This recurrent deletion shows mutual exclusivity with *TP53* loss, indicating it may be targetable with PARP inhibitors, and often encompasses additional DDR proteins, including *REV3L*, *ASCC3*, *ASF1A*, *MCM9*, and *MAP3K7* (Figure 3A) [122,123,124,125]. MMS22L derives its name from its identification in yeast screens for sensitivity to the alkylating agent methyl methanesulfonate (MMS) [126]. In CRISPR screens using the human hTERT-RPE1-*TP53* null cell line, all of the known DNA repair proteins in this deletion conferred some level of DNA damage sensitivity in vitro, particularly to alkylating agents, including cisplatin, MMS and MNNG (methylnitronitrosoguanidine) (Figure 3B) [127]. ASCC3 is part of the ASCC helicase complex that repairs alkylation damage, including that induced by MMS, in RNA and DNA [128]. REV3L is a subunit of DNA Polymerase Zeta that contributes to replication stress tolerance and lesion bypass, with an important role in ssDNA gap repair in HR-deficient backgrounds [129,130]. MCM9 is a component of a helicase complex, which includes MCM8 and HROB (also referred to as C17orf53 or MCM8IP), which was implicated in HR and interstrand crosslink repair [131,132,133,134,135]. The *ASF1A* gene is located within *MCM9*, and ASF1A was implicated in NHEJ [136,137]. The depletion of both ASF1A and its orthologue, ASF1B, also compromised the MMSL22-TONSL-mediated loading of RAD51 in HR [138]. Two papers have recently demonstrated that ASF1A loss conferred resistance to PARP inhibitors in *BRCA1*-mutant settings, where it was proposed to facilitate heterochromatin formation and inhibit resection, through interactions with RIF1 [139,140]. RIF1, a 53BP1 interactor, is a known suppressor of PARP inhibitor sensitivity in *BRCA1* deficiency (Figure 2D). ASF1A loss suppressed PARP inhibitor sensitivity in *BRCA1*-deficient cells to a similar extent as RIF1 loss, suggesting that ASF1A plays an early role in DSB repair pathway choice and could confound PARP inhibitor sensitivity in the context of the 6q deletion. Finally, the deletion of *MAP3K7*, also referred to as TAK1, reduced the expression of HR proteins and was shown to sensitize prostate cancer cells to the multi-CDK inhibitor dinaciclib or Olaparib [141]. MAP3K7 was also demonstrated to promote the degradation of AR protein levels in conjunction with a TNFa and NFKB-mediated inflammatory response, indicating that cancers with this Ch6q deletion may have elevated AR and reduced inflammation [142]. As the impact of the loss of this *MMS22L*-containing 6q locus containing multiple repair genes has not been assessed, it will be interesting to see if it confers clinical responses to PARP inhibitors and other DNA-damaging agents, such as platinum drugs, in future trials in CRPC.

The tripartite Ribonuclease H2 (RNASEH2A/B/C), endonucleolytically removes ribonucleotides from DNA to prevent replication stress, PARP trapping, and DNA damage [144,145]. Recurrent deletions of *RNASEH2B*, and to a lesser extent *RNASEH2A* and *RNASEH2C* (<1% deep deletions), occur in both primary and mCRPC patients, often co-occurring with *BRCA2* and *RB1* loss in the case of *RNASEH2B* [120,146]. The deletion of *RNASEH2B* sensitized PCa cell lines to PARP inhibition, as was observed previously in other cell types [145,146,147]. However, the concurrent loss of *RB1*, a frequent driver mutation in multiple CRPC subtypes (~10–15%), conferred resistance to PARP inhibition in *RNASEH2B*-deficient cells by elevating the E2F1-driven expression of *BRCA2* [83,147,148,149]. *RNASEH2B* loss does not impair HR and is synthetically lethal with *BRCA1* deficiency, indicating that the distinct mechanisms of PARP inhibitor sensitivity may be differentially affected by the genetic landscape of the particular cancer [145]. This also further points to the complexity of using single genes as biomarkers for DDR inhibitor use and the need for further analysis of diverse CRPC subtypes and their recurrent genomic rearrangements to identify genes that influence sensitivity or resistance to PARP inhibitors and other DDR-targeting agents.

PARP inhibitor efficacy may also be influenced by the TME, with hypoxia proposed to be both a sensitizer of HR-proficient cells to PARP inhibitors, as well as a barrier to PARP inhibitor efficacy in HRD cell lines from various cancers. Chronic hypoxia in cell culture impairs the transcription and translation of DDR genes, including RAD51, thereby reducing HR capacity and causing HR-dependent radiosensitization [150,151,152]. This effect of hypoxia on HR protein translation was demonstrated in multiple cell lines, where it created a contextual lethality that conferred a modest sensitization to PARP inhibitors in cell culture, as well as the killing of hypoxic tumor cells in mouse models in vivo [152]. These data suggest that PARP inhibitors could target chronically hypoxic tumors that are resistant to therapy by inducing a HRD state.

In other experiments under more moderate hypoxia, the PARP inhibitor efficacy decreased in both HR-proficient and HRD cell lines in vitro [153]. Interestingly, this effect was independent of HIF1α and associated with the reduced production of ROS, which cause ssDNA lesions, under hypoxia. Combining PARP inhibitors with the HAP Tirapazamine, which generates ssDNA damage, led to stronger cell killing in vitro and reduced tumor growth in multiple xenograft cancer models. While this approach has not been tested in PCa to our knowledge, it suggests that moderate hypoxia may impede the full potential of PARP inhibitors in PCa, and HAPs that augment the generation of DNA damage could be considered in this context.

## 5. Targeting DDR Kinases in CRPC Therapy

ATM, ATR, and DNA-PKcs play central roles in the DDR (Figure 2A,D) and the loss of any of these kinases provides numerous opportunities for synthetically lethal interactions [154]. Specific small molecule inhibitors for each kinase were developed and several inhibitors of ATR and DNA-PKcs are currently being tested in many cancer types as monotherapies or in combination therapies with genotoxic agents. In some cases, trials are taking advantage of *ATM* mutations that occur frequently across cancers and are synthetically lethal with ATR or DNA-PKcs deficiency [154].

Inhibitors of the ATR kinase sensitize cancer cells to replication stress and genotoxic agents have been extensively characterized in cell culture and pre-clinical mouse models, and were analyzed genetically through a number of siRNA and CRISPR screens [146,155,156,157,158,159,160,161,162,163,164]. Genetic sensitizers of ATR inhibitors include some genes that are recurrently mutated in CRPC, such as *ATM*, *BRCA1*, *BRCA2*, *RNASESH2B*, and *ARID1A* [122,158,165]. ATR inhibitors also sensitize cells to a number of genotoxic agents, including platinum drugs and topoisomerase inhibitors, both of which are used in a subset of CRPC treatments [166,167].

The direct testing of *ATM*-deficient PCa models have demonstrated sensitivity to ATR inhibitors that was further potentiated by the addition of PARP inhibitor, a combinatorial effect that has been observed in several cancer types [168,169,170,171]. The co-treatment of cells with both PARP and ATR inhibitors has now been explored in further detail and entered clinical trials for a number of cancers, including CRPC [172]. The combination of the ATR inhibitor AZD6738 and Olaparib is being tested in the Phase 2 TRAP trial (NCT03787680) on resistant mCRPC and AZD6738 is also being evaluated with Olaparib or Durvalumab (anti-PD-1 immunotherapy) in solid tumors including mCRPC (NCT03682289). Additional combination therapies between ATR inhibitors and genotoxic agents, including PARP inhibitors; topoisomerase I inhibitors; and gemcitabine, a cytidine nucleoside analog, are ongoing. The Phase 1/2 TRESR study is evaluating the ATR inhibitor RP-3500 alone or combined with Talazaporib or gemcitabine (NCT04497116) and another Phase 1/2 study is examining the ATR inhibitor ART0380 in combination with the topoisomerase 1 inhibitor irinotecan or gemcitabine (NCT04657068). The combination of ATR inhibitors and topoisomerase 1 inhibitors has shown promise in trials for small cell lung cancer that has included a small number of CRPC-NE patients. A recent Phase 1/2 trial on neuroendocrine cancers using ATR inhibitors in conjunction with sacituzumab govitecan (Trodelvy), an antibody–drug conjugate that couples an antibody for Trop-2 to the topoisomerase inhibitor SN-38, showed tumor regression in two CRPC-NE patients (NCT04826341) and this study is now being extended to include PARP inhibitor-resistant HRD tumors [173]. *BRCA1*-deficient cells that acquired PARP inhibitor resistance have shown increased dependence on ATR activity, suggesting that resistant HRD tumors may benefit from ATR inhibition [174,175].

Mutations in *RNASEH2B*, which were investigated in the context of PARP inhibitors in CRPC, have also been identified as sensitizers of ATR inhibition, topoisomerase 1/2 inhibitors, and gemcitabine through whole-genome CRISPR screens in other cell types [146,147,155,176]. As the loss of RNASEH2 function does not impair HR, it remains unclear what genomic features of CRPC may influence this strategy. For example, whether *RB1* loss, which rescued PARP inhibitor sensitivity in *RNASEH2B*-deficient cells, would also abrogate ATR inhibitor or genotoxic agent sensitivity in this background remains to be determined [147].

DNA-PKcs (encoded by *PRKDC*) is frequently upregulated in prostate cancer [177,178]. In addition to its role in DNA repair, DNA-PKcs plays a variety of transcriptional roles in AR-CRPC, where it was identified as a cofactor for the AR, and more recently, AR-Vs, and its high expression correlated with aggressive and metastatic tumors [177,179]. DNA-PKcs activity has been shown to regulate splicing and the expression of AR-Vs through the control of *RBMX* expression [179]. The KU70 subunit of DNA-PK has been reported to recognize Topoisomerase 1 cleavage complexes to facilitate enhancer-mediated transcription in breast and prostate cancer [180,181]. DNA-PK has been shown to regulate glycolysis in PCa through the phosphorylation of ALDOA and PKM2 and DNA-PK also has a poorly understood role in translation, where it was shown to be activated by several small RNAs and regulate the processing of the 18S rRNA [182,183].

Numerous DNA-PKcs inhibitors have entered clinical trials for a variety of cancer types, often in combination with RT or other DNA-damaging agents (recently reviewed in reference [37]). Some of these agents also target mTOR1 and 2, structurally related kinases that control cell growth in response to nutrient availability [184]. In CRPC, the dual DNA-PKcs/mTOR targeting agent CC-115 was tested with the ARSI Enzalutamide and is moving to Phase II trials following a successful Phase I safety assessment (NCT02833883) [185]. While DNA-PKcs has not yet been proven to be an effective target as a monotherapy in CRPC, it may prove to be valuable in the context of combination therapies in future studies. Pre-clinical data has shown that DNA-PKcs inhibitors could re-sensitize taxane-resistant cells and restore sensitivity to multiple genotoxic agents, including topoisomerase inhibitors and platinum drugs, and ongoing clinical trials are testing DNA-PKcs inhibitors with RT, PARP inhibitors, and CHK1 inhibitors in a variety of cancer types [37,186].

## 6. Generating Targeted DNA Damage with Radiopharmaceutical Therapy

Aside from targeting DDR signaling, active efforts to target DNA-damaging radionuclides to cancer cells are underway. Radionuclides can be used for either imaging or therapy depending on the type of radiation emissions intrinsic to the radioisotope. Beta particle-, alpha particle-, or Meitner–Auger-emitting radionuclides can cause single- and double-strand DNA breaks (SSBs and DSBs) and/or damage to critical organelles when delivered to cancer cells as radiopharmaceutical therapy (RPT) [187]. How these RPT agents compare to EBRT with respect to the extent of SSBs or DSBs is challenging given their heterogeneity of distribution within targeted tumors and the differences in dose rates, with radionuclides having much lower dose rates compared to EBRT.

The intrinsic properties of some radionuclides allow for tissue specific targeting. For example, the alpha particle-emitting radium-223 is a calcium mimetic that localizes to areas of bone turnover, including bone metastases. Radium-223 (^223^RaCl_2_, Xofigo) has been FDA-approved for use in patients with symptomatic bone-limited mCRPC, following the results of the Phase 3 ALSYMPCA (NCT00699751) trial which showed improved overall survival compared with placebo [188].

To target soft tissue lesions, therapeutic radionuclides need to be coupled to a tumor- or TME-selective targeting molecule. Prostate-specific membrane antigen (PSMA), which is highly expressed in prostate adenocarcinoma, was discovered in the 1990s and has been an attractive target for both imaging and therapy [189]. First, antibodies and, later, peptidomimetics that bind to the extracellular domain were developed and tested as imaging or therapeutic agents for this disease. This culminated in the FDA approval of the PSMA-specific, beta particle-emitting peptidomimetic lutetium-177 vipivotide tetraxetan (a.k.a. ^177^Lu-PSMA-617, Pluvicto) in 2022, following the pivotal Phase 3 VISION study (NCT03511664), which demonstrated an improved overall survival benefit when compared with the protocol-defined standard of care in patients with mCRPC who had progressed after first-line taxane therapy. Unsurprisingly, ^177^Lu-PSMA-617 is now being tested prospectively in earlier lines of therapy. For example, a Phase 3 study, PSMAfore, is comparing RPT to ADT change in mCRPC (NCT04689828), one Phase 2 study is evaluating ^177^Lu-PSMA-617 when combined with first line docetaxel in mCRPC (NCT04663997), and in a Phase 1/2 trial preoperatively (NCT04430192).

While these earlier-line treatments continue to be pursued in potentially less heterogeneous and aggressive prostate adenocarcinoma, additional studies are exploring combinations with other therapies, which may synergize with ^177^Lu-PSMA-617. One example is the randomized Phase 2 cohort trial of stereotactic ablative radiotherapy versus stereotactic ablative radiotherapy combined with ^177^Lu-PSMA-617 (NCT05560659) in patients with oligometastatic mCRPC. The PROQURE study is evaluating node-positive hormone-sensitive prostate cancer receiving ^177^Lu-PSMA-617 with definitive external beam radiotherapy and ADT (NCT05162573). Another trial, PSMAddition, is a Phase 3 trial evaluating the standard of care with or without ^177^Lu-PSMA-617 in metastatic hormone-sensitive prostate cancer (NCT04720157).

Because the predominant mechanism of cytotoxicity of ^177^Lu-PSMA-617, and RPT generally, is DNA damage, the combination with DDR inhibitors is a natural line of inquiry. For example, the Phase 1 LuPARP trial (NCT03874884) is evaluating the combination of ^177^Lu-PSMA-617 and the PARP inhibitor Olaparib. Investigators are also prospectively testing the DNA-PKcs inhibitor, peposertib, in a Phase 1/2 trial combined with ^223^RaCl_2_ with or without avelumab (PD-L1 immunotherapy) in patients with mCRPC (NCT04071236). As mutations in *ATM*, *BRCA2*, and many other DDR proteins that would be predicted radiosensitizers occur in CRPC with some frequency, future efforts may seek to take further advantage of genetic lesions and DDR inhibitors in RPT.

Beyond clinical studies, answers to other open questions are being pursued. For instance, personalized dosimetry, that is, modulating the activity administered to patients based on patient-specific features (e.g., tumor burden and estimated absorbed dose to tumor and organs at risk), has the potential to improve the therapeutic index of these agents by limiting both over- and underdosing. Derivatives of PSMA-targeting agents containing alpha particle-emitting radionuclides such as actinium-225 (^225^Ac), including ^225^Ac-PSMA-617, are being tested in Phase 1 studies (NCT04597411). Alpha particles exhibit high linear energy transfer, that is, deposit high energy over a short pathlength, and they cause several-fold more DNA damage than beta particles. In some studies, patients who were refractory to ^177^Lu-PSMA-617 demonstrated radiographic and biochemical responses to ^225^Ac-PSMA-617 [190]. Antibody-based PSMA-targeted RPT agents, which have distinct biodistributions and toxicity profiles from peptidomimetic agents, are also being explored (NCT04876651). If effective, these derivatives will surely be tested in combinations with other modalities or drugs, as have ^177^Lu-PSMA-617 and ^223^RaCl_2_. Other PSMA-targeted ligands, including the well-known PSMA-I&T, are also being coupled to imaging and therapeutic radionuclides for testing in prospective clinical trials. Another PSMA-targeted agent, ^18^F-rh-PSMA-7.3, was recently FDA-approved for imaging, and its therapeutic counterpart, ^177^Lu-rh-PSMA-10.1, is being clinically evaluated in a Phase 1/2 clinical trial (NCT05413850) [191,192].

## 7. Crosstalk between the Immune System and the DDR in CRPC

Immune checkpoint blockade (ICB) immunotherapy has garnered extensive interest, as targeting immune checkpoint receptors, such as PD-1, PD-L1, CTLA4, and LAG-3, effectively suppresses immunosuppressive pathways in the TME in many cancer types [193,194]. ICB aims to disrupt signaling between cancer cells and the immune system that inhibits T-cell proliferation and anti-tumor immune responses. The observation that RT and other DNA-damaging agents induce inflammatory responses, which include the induction of several of these receptors, has launched a large number of studies aimed at understanding how to best combine DDR activation and ICB (recently reviewed in reference [65]).

High TMB, MSI-H, or dMMR are the primary clinical indicators used to guide ICB use, presumably due to the need for neoantigens to drive the immune response [193,195]. Tumor heterogeneity and active nonsense-mediated mRNA-decay have also been shown to limit the capacity of immune responses to engage tumors with high TMB [196,197,198]. The low frequency of dMMR and APOBEC signatures in CRPC represents a potential limitation for ICB approaches [71,72]. Recent work has demonstrated that the sequencing of circulating tumor DNA could be used to identify hypermutation signatures and MMR defects in metastatic PCa, indicating that this could be a powerful screening tool for identifying ICB responders [75]. The genomic analysis of a cohort containing African patients revealed a higher overall TMB, including elevated mutational signatures for both HRD and dMMR, indicating potential opportunities for the use of PARP inhibitors and ICB [199].

Recent work has identified SYNCRIP, an RNA-binding protein implicated in splicing, as a suppressor of APOBEC3B activity in CRPC [77]. The deletion of SYNCRIP occurs frequently in CRPC and it was shown that this enhances APOBEC3B mutagenic activity that can drive resistance to AR-targeted therapies and promote lineage plasticity through the mutagenesis of a number of drivers, including *FOXA1*, in a pre-clinical model. The treatment of patient-derived explants with ARSIs upregulated APOBEC3B at the RNA and protein levels, suggesting that this could be part of a general response to AR-targeted therapies. APOBEC inhibitors, which are currently under development, could potentially be used to restrain lineage plasticity in future therapeutic approaches using ARSIs and other targeted therapies in CRPC [200,201,202,203,204,205]. How actionable APOBEC-mediated mutagenesis will be in CRPC, either to prevent resistance or enhance ICB, remains to be determined.

While inhibitors of CTLA-4 and PD-L1 have been tested in clinical trials for PCa, the only ICB therapy currently approved in mCRPC with high MSI or dMMR is the PD-1 inhibitor pembrolizumab (Keytruda) that blocks the binding of PD-1 on T-cells to its receptor PD-L1, which is upregulated in some cancer cells [193]. In localized prostate cancer, the loss of *BRCA2*, *RB1*, or the chromatin remodeler *CHD1*, is correlated with *PD-L1* expression and have been proposed as patient selection markers for ICB use [206]. However, whether this also applies to mCRPC remains to be determined. As a subset of CRPC patients appear to benefit from anti-PD-1 in unselected patient populations, a large number of clinical trials are ongoing with pembrolizumab and other ICB therapies (ongoing clinical trials were recently reviewed in reference [207]). These include testing patients with HR-proficient and HRD tumors, as well as combination therapies with PARP inhibitors, ARSIs, and RPT. One Phase 2 trial is comparing patients with mCRPC randomized to ^177^Lu-PSMA-617 or ^177^Lu-PSMA-617 with ipilimumab (anti-CTLA-4) and nivolumab (anti-PD-L1) (NCT05150236), and another Phase 1b study is assessing the safety and efficacy of ^177^Lu-PSMA-617 combined with pembrolizumab (NCT03805594). Such trials are of interest in light of preclinical work showing that RPT may enhance ICB in otherwise immunologically cold tumors [208].

Combining ICB with RT, ATR, DNA-PKcs, and PARP inhibitors has been shown to improve outcomes in some cancer types, in large part due the activation of innate immune responses driven by the cGAS-STING pathway [209,210,211,212,213,214,215,216,217,218]. The cGAS protein senses extrachromosomal DNA that can arise from DNA damage and activates the endoplasmic reticulum protein STING through the production of 2′3′-cGAMP [219]. STING activates downstream transcriptional activators, including IRF3 and NFKB, to activate antiviral inflammatory responses. Mutations in *RNASEH2B*, which occur frequently in CRPC, are associated with Aicardi–Goutieres syndrome, a hereditary inflammatory disease, and similar mutations occur in multiple cancers and have been correlated with inflammatory gene signatures, suggesting that these tumors may respond more readily to ICB [146,220,221]. STING agonists have been shown to improve ICB responses in preclinical CRPC models and have entered clinical use for a number of cancer types [222,223,224].

Recently, targeting B7-H3 (PD-L3; CD274), a member of the PD-L1 family, as well as Myeloid Derived Suppressor cells (MDSCs), has been demonstrated to overcome immunosuppression in CRPC, and other cancer types, in preclinical models [225,226]. The expression of B7-H3 was high in many PCa patient samples and cell lines, notably, higher than PD-L1 (B7-H1), and it correlated with adverse disease events [227,228]. The modulation of B7-H3 levels in multiple cell types, including *PTEN-TP53*-deficient CRPC, impaired tumor growth [229]. As PD-L1 expression is activated by RT and other DNA-damaging agents, it will be of interest to determine if B7-H3 levels can be similarly manipulated for clinical gain, for example by RPT or ATR/PARP inhibitors. Multiple strategies have been proposed to target the MDSC cell population that suppresses T-cell responses, alone or in combination with ICB, including the use of multi-kinase inhibitors, inhibitors of CXCL5-CXCL2 or co-targeting the MNK1/2 and AKT pathways [230,231]. A clinical trial in ARSI-resistant mCRPC using a combination of Enzalutamide and a CXCR2 inhibitor (AZD5069) showed promising results in a subset of patients, supporting this as a therapeutic strategy for further development (NCT03177187) [232]. Again, whether any of these strategies targeting MDSC-mediated immunosuppression will, like ICB, further benefit from DDR inhibitors or RPT remains to be tested in future work.

## 8. Targeting the Hypoxic TME in CRPC Therapy

Hypoxia contributes to genomic instability in several tumor types and is associated with high rates of TMB. For instance, hypoxic PCa tumors showed the loss of PTEN function, increased levels of chromothripsis (clustered chromosomal rearrangements), and shorter telomeres in localized PCa [233]. As hypoxia is a barrier to RT, chemotherapies, and immunotherapy, multiple strategies are being employed to directly exploit hypoxia in cancers. In PCa, two HAPs, TH-302/evofosfamide and CP-506, are being tested in clinical trials in patients with PCa. Evofosfamide and CP-506 are DNA-alkylating agents under low oxygen (<0.5% O_2_) conditions and both reduce tumor growth in a variety of preclinical models, including PCa [234,235,236,237]. CP-506 is being tested in combination with carboplatin or a selection of ICB agents (NCT04954599) and Evofosfamide is being tested with ICB (Ipilimumab/anti-CTLA4) (NCT03098160) in a variety of solid tumors, including PCa.

CRPC-NE is a form of lethal prostate cancer that is resistant to many types of therapy and shares some features with CRPC-SCL [22,238]. The homeodomain TF ONECUT2 (OC2) was recently shown to be a master regulator of neuroendocrine cancer, including CRPC-NE, and additional data have shown that the loss of *RB1* is sufficient to increase its expression in CRPC models [239,240]. OC2 depletion in PC3 cells impairs hypoxia-mediated transcriptional programs and drove NE differentiation that was augmented by hypoxia [239]. OC2 regulated HIF1α chromatin occupancy by upregulating SMAD3 expression and OC2 depletion impairs tumor growth in xenografts. OC2 expression has been correlated with high hypoxia in CRPC-NE, and multiple patient-derived xenograft (PDX) models were sensitive to treatment with Evofosfamide. Together, these data suggest that OC2 expression in CRPC-NE may serve as a marker for the use of treatments that take advantage of HAPs and other therapeutic approaches to exploit low oxygen, providing strategies to augment RT and DDR inhibitors that are impaired by hypoxia.

## 9. Emerging Targets in CRPC

**Figure 4:**
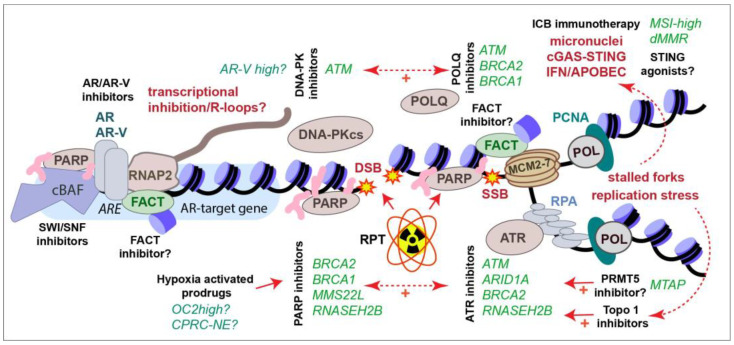
Summary of current and emerging therapies to treat CRPC. Selected genetic alterations (green italics) that sensitize to particular therapies (black bold) are shown. See text for full details.

A number of small molecule inhibitors targeting DDR components and chromatin regulators are being used in research and, in some cases, entering clinical trials. Here, we describe in brief a selection of their targets and their potential utility in CRPC treatment. Several are highlighted in Figure 4 in the context of previously described therapies.

### 9.1. SWI/SNF

Genes that encode members of the SWI/SNF nucleosome remodeling complexes are frequently mutated in many cancer types, including PCa [241]. SWI/SNF complexes have been shown to localize to DNA damage sites and its deficiency sensitizes cells to a number of DNA-damaging agents [242]. SWI/SNF has been implicated in NHEJ, HR, NER, and MMR, although its precise function in these pathways remains unclear [242,243]. Three major subtypes of SWI/SNF complexes have been identified that contain overlapping and unique subunits, and each of these complexes contains one of two ATPase subunits, SMARCA2 (BRM) and SMARCA4 (BRG1) [244]. The depletion of both SMARCA2 and SMARCA4 activity globally reduces chromatin access and leads to increased levels of R-loops, RNA–DNA hybrids, which can cause DNA damage and genomic instability [245,246]. Targeting both of these enzymes, as well as an additional SWI/SNF protein, PBRM1, using the AU-15330 PROTAC in PCa, was demonstrated to have preferential effectiveness in enhancer-addicted AR-dependent PCa [247]. SMARCA4/BRG1 was also identified as a selectively essential gene in *PTEN*-depleted cells in a CRISPR screen [248]. As the loss of *PTEN* occurs in up to 50% of CRPCs, and across all subtypes, this may be another vulnerability that can be exploited with agents that target SWI/SNF. The further expanded use of agents targeting SWI/SNF in CRPC may be achievable with combination therapies that exploit R-loop-mediated DNA damage or additional genetic interactions, but this remains to be experimentally determined. Currently, no agents directly targeting SWI/SNF have entered clinical trials, but given the large amount of activity in this space, this will likely change in the near future.

### 9.2. POLQ

Recently, several inhibitors were identified for POLQ, which is required for TMEJ, that can serve as a backup pathway for DSB repair (Figure 2D) [41,249,250,251,252]. POLQ loss is synthetically lethal with HRD, as well as with the loss or inhibition of *ATM* or *ATR*, providing another route to target a subset of CRPCs, including those that become resistant to PARP inhibitors [249,250,253,254,255]. Although not in PCa, several studies have combined POLQ inhibitors with DNA-PKcs inhibitors and observed the increased killing of p53-deficient cells, as well as enhanced radiosensitivity [256,257]. POLQ inhibition also enhances PARP inhibitor effectiveness and activates the cGAS-STING pathway in an HRD pancreatic adenocarcinoma mouse model, suggesting that it may have applications in ICB immunotherapy, in which STING agonists are also under investigation [253]. While POLQ inhibitors have not been explored in PCa to our knowledge, they may be valuable reagents to investigate for future combination therapy approaches involving NHEJ (DNA-PKcs inhibitors), PARP inhibitors, RT, or ICB, as well as mutations in selected DDR genes. Phase I and II trials are ongoing with several POLQ inhibitors in metastatic solid tumors (NCT05898399, NCT04991480, NCT06077877, NCT05687110).

### 9.3. PRMT1

Protein arginine methyltransferase 1 (PRMT1) has been implicated in cell cycle progression, DNA repair, innate immunity, and RNA processing and proposed as a therapeutic target in several cancer types [258,259,260]. Its precise functions in the DDR remain unclear but it has been shown to methylate key DSBR regulators, BRCA1, MRE11, and 53BP1 and influence DSBR pathway choice [261,262,263,264,265,266]. Moreover, PRMT1 loss in mice caused increased levels of genomic instability and HRD [267]. Multiple small molecule inhibitors of PRMT1 have been developed, the first of which has entered clinical trials for a number of tumor types (NCT03666988) [259]. *PRMT1* expression is upregulated in advanced PCa and it has been identified as a regulator of AR-V7 in a whole-genome CRISPR screen [268,269]. The depletion of *PRMT1* reduces the occupancy of AR at its target genes, an effect that has also been observed following treatment with the metabolite spermine, which has been shown to inhibit PRMT1 [269,270]. Using a LNCaP-derived cell line with higher AR expression (LNCaP/AR-Enh) it was demonstrated in xenografts that co-targeting AR and PRMT1 was an effective therapeutic approach [269]. Collectively, these data suggest that targeting PRMT1 in combination with ARSIs may be an effective strategy for CRPC-AR and that combining PRMT1 inhibitors with genotoxic agents may be warranted.

### 9.4. PRMT5

Protein arginine methyltransferase 5 (PRMT5) targets proteins involved in many cellular processes, influences AR signaling and regulates the DDR through direct and indirect mechanisms, leading to radio- and chemosensitivity (recently reviewed in references [271,272]). PRMT5 loss impairs HR and enhances the toxicity of PARP inhibitors in other cancer types [273]. PRMT5 loss is synthetically lethal with *MTAP* deficiency, which occurs frequently in many cancer types, including PCa, providing an additional rationale for the use of PRMT5 inhibitors in selected patient populations [274]. PRMT5 has also been identified as a suppressor of DNA damage in CRISPR screens and subsequent analysis has demonstrated that PRMT5 inhibitors destabilize ATM in cell cultures, suggesting that it could be used in combination with a variety of DNA-damaging agents, such as ATR or PARP inhibitors, in future therapies [275]. PRMT5 inhibition can also improve ICB outcomes through the augmentation of cGAS-STING responses and PD-L1 expression [276,277]. Two recent reports have demonstrated that PRMT5 inhibition impairs DNA repair, enhances radiosensitivity in multiple CRPC cell lines, and prevents RT-induced neuroendocrine differentiation and tumor growth in CRPC-AR tumor models [278,279]. PRMT5 has also been identified as a suppressor of gemcitabine toxicity in whole-genome CRISPR screens of pancreatic cells [280]. Thus, PRMT5 inhibitors could potentially be useful in treating both castration-sensitive PCa and CRPC and could potentially be enhanced by selection for *MTAP* loss or combination therapies. Currently, more than 10 Phase 1/2 trials are ongoing with PRMT5 inhibitors in a variety of cancer types, in some cases, utilizing *MTAP* loss for cohort selection (https://clinicaltrials.gov/ (accessed on 10 December 2023)).

### 9.5. FACT

The Facilitates Chromatin Transcription (FACT) complex, composed of SSRP1 and SPT16, plays a key role in nucleosome dynamics during transcription, DNA replication, and DNA repair (reviewed in reference [281]). The elevated expression of FACT subunits is often observed in cancer, in some cases correlating with poor clinical outcomes [282]. Both FACT subunits have been identified as common essential genes, indicating that many cancers depend on maintaining FACT function (Depmap.org). The drug CBL0137/Curaxin is a non-mutagenic DNA intercalator that has been shown to trap FACT on chromatin, leading to transcriptional alterations, including the activation of the TP53 response [283]. CBL0137 has been demonstrated to display anti-tumor efficacy as a single agent in glioblastoma mouse models, enhance radiotherapy and histone deacetylase inhibitor responses in CNS tumors, as well as enhance the responses to cisplatin in small-cell lung cancer [284,285,286,287,288,289]. In acute myeloid leukemia cell lines, CBL0137s toxic effects were limited to those with *TP53* mutations, suggesting its efficacy may be optimal in TP53-proficient cells [290]. FACT interacts with the TONSL-MMS22L complex that is important for HR and frequently compromised in CRPC, as discussed earlier (Figure 4) [117,118,119,120]. Consistent with this, CBL0137 was shown to impair HR and enhance PARP inhibitor and carboplatin efficacy in serous ovarian carcinoma [291]. The depletion of FACT or treatment with CBL0137 results in the activation of the IFNg response, consistent with previous reports that it is required for endogenous and exogenous viral silencing [292,293,294]. Notably, CBL0137 has been identified in a screen to bypass anti-PD-1 ICB suppression by ADAR1, an RNA-editing enzyme that attenuates antiviral responses [295]. This suggests that it may be interesting to try CBL0137 in combination with ICB, which can be enhanced by innate immune activation, including IFNg [296]. Finally, FACT has been demonstrated to mediate adaptation to hypoxia and CBL0137 impaired tumor growth and enhances the effects of an anti-angiogenic drug, bevacizumab, in a hepatocellular carcinoma model [297]. While the effects of CBL0137 have not been reported in PCa, it has entered clinical trials for a number of other cancer types, and its potential to enhance genotoxic agents, impair transcription, sensitize hypoxic cells, and improve DDR inhibitor and ICB responses may be useful to explore in future work (NCT01905228, NCT02931110, NCT03727789, NCT05498792, NCT04870944).

### 9.6. The Tousled-Like Kinases (TLKs)

The Tousled Kinase (TSL) was first identified in plants and later the mammalian Tousled like kinases, TLK1 and TLK2, were connected to the DDR when TLK1 was identified as a CHK1 substrate [298,299,300,301]. TLK activity is required for DNA replication and chromatin maintenance, primarily through the regulation of ASF1A and ASF1B [301]. These chaperones facilitate the incorporation of H3/H4 heterodimers during replication, transcription, and DNA repair [301]. TLK1 was shown to regulate additional DDR factors, including RAD9 and RAD54L, which play roles in TMEJ and HR, respectively [302,303,304,305,306]. The depletion of TLK1/2 leads to replication stress and DNA damage that could be enhanced by ATR, CHK1, or PARP inhibitors in several cell lines in vitro [307]. This is accompanied by the activation of cGAS-STING-dependent innate immune responses, including TNFa and IFNg, similar to what has been observed for *ASF1A* depletion that has been demonstrated to enhance anti-PD-1 ICB immunotherapy via TNFa induction [308,309]. The short hairpin-mediated depletion of *TLK2* in breast cancer xenograft models or treatment LNCaP (CRPC-AR) xenografts with TLK1 inhibitors thioridazine or J54-impaired cancer growth, an effect that was enhanced by anti-hormone treatment in both cases [310,311,312]. Additional TLK inhibitor leads based on indirubin E804 have also recently been reported and shown to inhibit ASF1A phosphorylation, impair DNA replication, and induce replication stress and DNA damage [313]. Thioridazine is an antipsychotic medication with a number of potential targets that has been evaluated in a number of clinical trials. However, its effects are unlikely to be restricted to the inhibition of TLK and no clinical trials have employed it for this reason to our knowledge. Existing evidence suggests that the development of more specific and potent inhibitors may be valuable for several cancer types, including CRPC, and that TLK inhibitors could be used in combination with a number of anti-cancer agents including ARSIs, DDR inhibitors, and ICB.

## 10. Conclusions

There are several opportunities to exploit the DDR in CRPC therapy, taking advantage of recurring genetic lesions or combination therapies (Figure 4). The AR remains a key target in PCa therapy and the continued development of new small molecules to target the AR and AR-Vs may improve future approaches and lend themselves to rational combination therapies, including with DDR-targeting agents. Tumor hypoxia has been associated with worse outcomes at every stage of prostate cancer disease progression and remains a significant barrier to radio- and chemotherapies, including PARP inhibitors. The use of HAPs, and possibly FACT inhibitors, may allow investigators to take advantage of this prominent feature of the PCa TME.

While evidence for HDR and other DDR mutations that facilitate PARP inhibitor use continues to grow, the generally low TMB of CRPC has limited immunotherapy to a subset of patients, with little evidence of clinical efficacy thus far. The identification of new targets, such as B7-H3 and MDSCs, integrated with the manipulation of the DDR, for example via PARP or ATR inhibitors or STING agonists, may determine if more CRPC patients can benefit from immunotherapy approaches in future trials. In addition, the generation of additional syngeneic models of CRPC could aid in the analysis of the interplay between the DDR and immune responses.

The RPT field has undergone a renaissance following recent regulatory approvals based on efficacy in clinically meaningful endpoints; namely quality of life and/or overall survival. Long-known agents are being tested in prospective studies to obtain regulatory approvals. Novel agents and tweaks of existing agents to improve their pharmacokinetic profiles have been synthesized and tested preclinically with the goal of clinical translation. Many opportunities to combine RPT with DDR modulators are waiting to be further explored and the results of open clinical trials will help clarify the best use of these agents.

While agents such as PARP inhibitors appear to have low toxicity based on current clinical information, the variable effects of individual agents or therapeutic combinations on normal tissue toxicity, particularly in diverse genetic backgrounds, will need to be assessed in future studies. Defining the optimal order of treatments to ensure the safest and most efficacious outcomes for patients will also be of high priority in future pre-clinical experiments and clinical trials.

## Abbreviations

ADT	Androgen deprivation therapy
Alt-NHEJ	Alternative non-homologous end joining
APOBEC	Apolipoprotein B mRNA editing enzyme, catalytic polypeptide
AR	Androgen receptor
AR-V	AR-variants
ARE	Androgen response element
ARSI	Androgen receptor signaling inhibitor
BER	Base excision repair
BRCA	Breast cancer associated
CE3	Cryptic exon 3
cNHEJ	Classical non-homologous end-joining
CRISPR	Clustered regularly interspaced short palindromic repeats
CRPC	Castration resistant prostate cancer
CRPC-AR	CRPC-androgen receptor subtype
CRPC-NE	CRPC-neuroendocrine subtype
CRPC-SCL	CRPC-stem cell like subtype
CRPC-WNT	CRPC-WNT dependent subtype
DBD	DNA binding domain
DDR	DNA damage response
dMMR	Mismatch Repair deficiency
DSB	Double-strand break
DSBR	DSB repair
dsDNA	Double-strand DNA
EBRT	External beam radiotherapy
HAP	Hypoxia activated prodrug
HD	Hinge domain
HIF	Hypoxia inducible factor
HR	Homologous recombination
HRD	Homologous recombination deficiency
ICB	Immune checkpoint blockade
LBD	Ligand binding domain
mCRPC	Metastatic CRPC
MMR	Mismatch Repair
MMS	Methyl methanesulfonate
MNNG	Methylnitronitrosoguanidine
MRN	MRE11-RAD50-NBS1
MSI	Microsatellite instability
NTD	N-Terminal domain
PARP	Poly (ADP ribose) polymerase
PCa	Prostate cancer
PSA	Prostate specific membrane antigen
RNASEH2	Ribonuclease H2
ROS	Reactive oxygen species
RPT	Radiopharmaceutical therapy
RT	Radiotherapy
SBS	Single base substitution
SSB	Single-strand break
SSBR	SSB Repair
ssDNA	Single-strand DNA
TF	Transcription factors
TLK	Tousled-like kinase
TMB	Tumor mutational burden
TME	Tumor microenvironment
TMEJ	Polymerase theta-mediated end-joining
